# Comparison of mini-open versus all-arthroscopic rotator cuff repair: retrospective analysis of a single center

**DOI:** 10.11604/pamj.2020.37.132.19491

**Published:** 2020-10-06

**Authors:** Yaman Karakoc, Ïsmail Burak Atalay

**Affiliations:** 1 Department of Orthopaedics and Traumatology, Oncology Training and Research Hospital, Ankara, Turkey

**Keywords:** Arthroscopy, shoulder joint, repair, rotator cuff tear, all-arthroscopic rotator cuff surgery, mini-open rotator cuff surgery

## Abstract

**Introduction:**

the aim of this study was to compare the impacts of all-arthroscopic repair and mini-open repair for rotator cuff tendon tear on post-operative pain, shoulder joint range of motion and physical function.

**Methods:**

the study was a retrospective comparative analysis of rotator cuff repair between January 2013 and January 2018. The patients included in the study were enrolled into all-arthroscopic surgery or mini-open surgery groups. Patients were assessed with a 10mm visual analog scale for pain in the 7^th^ day post-operatively. The physical function was assessed with Quick Disabilities Arm Shoulder and Hand (DASH) questionnaire at 12^th^ month. The flexion and abduction ROM of the involved site were measured preoperatively and 12 months after the surgery.

**Results:**

a total of 40 patients were included in the study. The mean age of the all-arthroscopic surgery group was significantly lower than the open surgery group (46.9±6.9 vs. 52.45±4.0 years). While no complication was seen in the arthroscopic group, 5 patients had superficial infection in the open surgery group. The patients in the all-arthroscopic surgery group experienced significantly less pain in the 7^th^ day of the surgery. Improvement in Quick Dash score and shoulder flexion after surgery were significantly higher in the all-arthroscopic surgery group. None of the patients needed revision surgery in both groups.

**Conclusion:**

according to the results of this study, arthroscopically operated patients with rotator cuff tear had less pain in the first week after surgery. Patients in this group had better shoulder flexion and function in long-term follow-up with no post-operative complication.

## Introduction

The management of a rotator cuff tear is an area of debate because various factors such as etiology of the tear (degenerative or traumatic) being symptomatic or asymptomatic, functionality (compensated or decompensated) may influence the surgeon´s decision [1-3]. To date, various surgical techniques including open, mini-open, all-arthroscopic techniques, have been previously performed by the surgeons for rotator cuff tear repair (RCTR) [3-5]. However, the outcomes of these studies are debatable. The size and shape of the tear, muscle fiber atrophy, and tendon retraction may influence the outcome of the surgery [3-5]. Surgeons have also investigated whether the surgical technique may affect RCTR outcome [3,4]. Although mini-open repair has been widely preferred for RCTR with good to excellent results in most of the patients, with the evolution of arthroscopic techniques, there is a tendency for performing RCTR arthroscopically [3-5]. Several retrospective and a few prospective comparison studies have evaluated the outcome of these surgical techniques with regards to pain, joint mobility, complications and surgery time [6-12]. However, most of the studies focused on early or long term post-operative pain with a relatively short period of follow up (6 weeks to 6 months) [6-12]. Long term joint range of motion (ROM) and physical function shave not been compared adequately. Accordingly, the aim of this study was to compare the outcomes of two popular surgical techniques, all-arthroscopic and mini-open, for RCTR regarding the early post-operative pain and long term shoulder joint ROM and physical function in a tertiary center.

## Methods

The study was retrospective comparative analysis of rotator cuff repair in Dr. Abdurrahman Yurtaslan Ankara Oncology Training and Research Hospital between January 2013 and January 2018. During this period a total of 92 patients were operated for rotator cuff tear. The patients included in the study were enrolled into all-arthroscopic surgery or mini-open surgery groups according to the surgeon who performed the operations. Patients complaining with shoulder pain and who were diagnosed with full thickness rotator cuff tear according to the magnetic resonance images were included in the study. Patients without full thickness tear, patients with additional shoulder disease (adhesive capsulitis, inflammatory arthritis) and those who did not complete regular follow up visits were excluded. Since the study was conducted retrospectively and the data were obtained from the patients files, it does not require obtaining written informed consent. The patients who had undergone arthroscopic operation were applied general anesthesia in lateral decubitus position. The inner parts of the joint were visualized through anterior and posterior portals. The degenerative changes, synovial hypertrophy and rotator cuff rupture remnants were removed. Then, subacromial space is reached via lateral portal. In case of inflammation and hypertrophic bursa, subacromial bursectomy was done from lateral portal in order to obtain a clear view of the tear. Subacromial decompression is obtained by anterolateral acromioplasty. The humeral attachment point was abraded without decortication. The 5 mm titanium suture anchors were placed to the greater tuberosity with single row technique. The patient´s undergone mini-open technique were applied general anesthesia in lateral decubitus position. Posterior and anterior portals were opened respectively and diagnostic shoulder arthroscopy was performed. After visualization of the inner parts of the joint, the degenerative changes and synovial hypertrophy were removed and rotator cuff tear were investigated. Then, subacromial space is reached via lateral portal. In case of inflammation and hypertrophic bursa, subacromial bursectomy was done from lateral portal. The procedure was started with 4-5 mm lateral transvers incision. The subacromial space was reached after the deltoid muscle fibers were dissected. Afterwards, the degenerated parts of the greater tuberosity were revitalized with scraper. The tendons were repaired using 5 mm titanium suture anchors. Patients were assessed with a 10 mm visual analog scale for pain in the 7^th^ day post-operatively. The physical function was assessed with Quick Disabilities Arm Shoulder and Hand (DASH) questionnaire at 12^th^ month. The flexion and abduction ROM of the involved site were measured with a simple goniometry preoperatively and 12 months after the surgery. The complications were noted during follow up period. The statistical analysis of the data was performed using Statistical Package for the Social Sciences version 16.0 (SPSS INC., IL, USA). The data are given as mean ± standard deviation or n, (%). After checking the normal distribution with Kolmogorov-Smirnov test, Student´s t-test was used for between-group comparisons for the numeric variables. Chi square test was used for the comparisons of categorical variables. Before surgery and after-surgery data was compared using Paired t-test. A p value less than 0.05 was considered statistically significant.

## Results

A total of 40 patients (20 in all-arthroscopic surgery group and 20 in mini-open surgery group) were included in the study. The demographic data and clinical features of the patients are summarized in Table 1. The mean age of the all-arthroscopic surgery group was significantly lower than the open surgery group (46.9±6.9 vs. 52.45±4.0 years). The gender, involved site and mean follow up were not statistically different between the two groups (P>0.05 for all). There was no complication in the arthroscopically treated group, none the less 5 patients had superficial infection in the open surgery group. The patients in the all-arthroscopic surgery group experienced significantly less pain in the 7^th^ day of the surgery. The Quick Dash score and shoulder ROM (flexion and abduction angles) measured before and 12 months after surgery and the significance level of the comparison of these values are shown in Table 2. According to these values, the mean pre-operative Quick Dash score was lower and shoulder flexion and abduction angles were higher in the all-arthroscopic surgery group. Therefore, we calculated the delta values of each parameter and compared the outcome. According to these values the improvement in Quick Dash score and shoulder flexion after surgery were significantly higher in the all-arthroscopic surgery group. None of the patients needed revision surgery in both groups.

**Table 1:** clinical and demographical features of the groups

Variables	All-arthroscopic surgery(N=20)	Mini-open surgery(N=20)	P-value
**Age (yr)**	46.9 ± 6.9	52.45 ± 4.0	0.004
**Gender**			0.113
Male	7 (35)	12 (60)	
Female	13 (65)	8 (40)	
**Involved side**			0.525
Right	12 (60)	10 (50)	
Left	8 (40)	10 (50)	
**Follow-up (month)**	25.0 ± 7.7	25.9 ± 7.7	0.730
**Complication**			0.017
None	0	15 (75)	
Superficial infection	0	5 (25)	
**Postoperative VAS(7th day)**	3.35 ± 1.1	4.80 ± 1.3	0.001

The data are given as mean ± standard deviation or n, (%); Student's t-test was used for between group comparisons for the numeric variables; Chi square test was used for the comparisons of categorical variables.

**Table 2:** preoperative and postoperative data of the groups

Variables	Arthroscopic surgery (20)	Open surgery(N=20)	P value
**Quick Dash score**			
Preoperative	63.1 ± 3.3	71.0 ± 2.8	**0.006**
Postoperative	24.7 ± 3.0	29.3 ± 2.8	**<0.001**
Delta Value	38.4 ± 2.5	41.6 ± 2.1	**0.030**
**Shoulder flexion**			**<0.001**
Preoperative	91.7 ± 11.7	76.8 ± 6.0	
Postoperative	157.5 ± 16.1	134.1 ± 10.1	**<0.001**
Delta Value	65.7 ± 15.0	57.3 ± 10.4	**0.046**
**Shoulder abduction**			
Preoperative	88.8 ± 11.0	72.6 ± 6.4	**<0.001**
Postoperative	152.2 ± 16.7	129.5 ± 10.9	**<0.001**
Delta value	63.4 ± 12.7	56.9 ± 11.9	0.105

The data are given as mean ± standard deviation; Student's t-test was used for between group comparisons.

## Discussion

The current study comparing the outcome of mini-open and all-arthroscopic surgical techniques for RCTR revealed that the arthroscopically operated patients had lower level of pain in the early post-operative period the physical function and shoulder flexion were better improved with lower incidence of post-operative complications in the all-arthroscopic surgery group. In the previous literature, several studies have been conducted in order to compare the outcome of mini-open and all-arthroscopic surgeries, and they reported similar outcomes [6-12]. In the meta-analysis on randomized controlled trials comparing the outcome of arthroscopic and mini-open rotator cuff repair, conducted by Ji *et al*. [2] the authors founded no differences with regards to surgery time, functional outcome score, VAS pain score and ROM between these two techniques. Recently, in their study, Bayle *et al*. [13] compared open and arthroscopic RCTR in 87 patients with a 12 months follow-up period. The patients were assessed with American shoulder and elbow surgeons (ASES) and simple shoulder value (SSV) for functional assessment, and ROM of shoulder joints at 3, 6 and 12 months after surgery. Additionally, the patients were also evaluated with magnetic resonance imaging (MRI) or arthro-computed tomography scan in order to investigate tendon retraction and fatty degeneration at follow-up. The authors concluded that, there was no significant difference in terms of function and ROM between the arthroscopic and open RCTR. In the current study, we compared arthroscopic and mini-open surgery techniques. The pain was evaluated at the 7^th^ day after surgery, and patients treated with mini-open surgery experienced significantly higher pain. Despite the findings in the study by Bayle *et al*. [13], flexion ROM and functional assessment score did improve more prominently in our study. In addition, both techniques were performed with the same surgeon in our study. However in the study by Bayle *et al*. [13], the techniques were performed by two different surgeons. The variations on experiences of the surgeons on each technique might explain the difference. In a previous study, Kasten *et al*. [8] prospectively compared the mini-open and all-arthroscopic repair of the supraspinatus tendon. They included totally 34 patients with similar MRI findings.

The patients in arthroscopy group underwent double-row repair while in mini-open group the patients received transosseous repair. They found that, although arthroscopically treated group had less pain in the first week, the patients in the mini-open group had less pain between the 4^th^ and 8^th^ weeks. The shoulder ROM and physical function which was evaluated with Constant-Murley score were similar in both groups. In our study, the patients were followed for mean 12 months and we found improved flexion and physical function evaluated with Quick Dash score. The difference in outcome might be partly explained with different functional assessment questionnaires and our long period follow-up. The complication rates after arthroscopic versus open rotator cuff repair were compared by Baker *et al*. [14]. The authors reported that patients undergoing open surgery had higher risk of any adverse event, higher risk to readmission for surgery within 30 days after surgery. However, the patients undergoing open surgery were older and had higher incidences of comorbid diseases. In our study, none of the patients needed for revision surgery, 5 of 20 patients had superficial infection in mini open surgery. This study has several limitations. Although the study is performed in a long period of time, the number of the patients included in this study might be relatively low. The patients were not randomized into two different surgery groups. Accordingly the preoperative age, ROM and functional status were differing between groups. To overcome this issue we used delta analysis (the difference between pre- and post-operative values) for comparison. Both surgical techniques were performed by the same surgeon who is experienced in shoulder surgery as such this fact allows to make a proper comparison. Finally, we did not compare the tear size, retraction stage of the patients, because we did not use an imaging modality during follow-up.

## Conclusion

According to the results of this study, arthroscopically operated patients with rotator cuff tear had less pain in the first week after surgery and better shoulder flexion and function in long-term follow-up with no post-operative complication. Our results may explain the tendency of the surgeons through arthroscopic surgery.

### What is known about this topic

- *Several studies have been conducted in order to compare the outcome of mini-open and all-arthroscopic surgeries for shoulder, and they reported similar outcomes*.

### What this study adds

- *In our study arthroscopically operated patients with rotator cuff tear had less pain and better shoulder range of motion in long-term follow-up*.
